# Short-term pretreatment with deferoxamine enhances the in vivo vascularization capacity of nanofat seeded onto dermal substitutes

**DOI:** 10.17179/excli2026-9365

**Published:** 2026-05-07

**Authors:** Valeria Pruzzo, Francesca Bonomi, Ettore Limido, Andrea Weinzierl, Yves Harder, Matthias W. Laschke

**Affiliations:** 1 Institute for Clinical and Experimental Surgery, Saarland University, PharmaScienceHub (PSH), 66421 Homburg, Germany; 2 Department of Surgery, Ospedale Beata Vergine Mendrisio, Ente Ospedaliero Cantonale (EOC), 6850 Mendrisio, Switzerland; 3 Department of Plastic Surgery and Hand Surgery, University Hospital Zurich, 8006 Zurich, Switzerland; 4 Department of Plastic, Reconstructive, and Aesthetic Surgery and Hand Surgery, Centre Hospitalier Universitaire Vaudois (CHUV), 1011 Lausanne, Switzerland; 5 Faculty of Biology and Medicine, University of Lausanne (UNIL), 1011 Lausanne, Switzerland

**Keywords:** angiogenesis, biocompatibility, deferoxamine, dermal substitute, inflammation, nanofat

## Abstract

Insufficient early vascularization remains a major limitation for the successful integration of implanted dermal substitutes. To overcome this challenge, nanofat has recently been introduced as a promising fat derivative for implant seeding. The present study investigated whether short-term ex vivo pretreatment with the hypoxia-mimetic agent deferoxamine (DFO) can further enhance the in vivo vascularization capacity of nanofat. Nanofat from green fluorescent protein (GFP)^+^ donor mice was pretreated for 1 h with DFO (1 mM) or vehicle and subsequently seeded onto collagen-glycosaminoglycan-based dermal substitutes, which were implanted into dorsal skinfold chambers of syngeneic GFP^-^ recipient mice. Implant vascularization, microhemodynamics, tissue integration and inflammatory response were assessed over a 14-day period using intravital fluorescence microscopy, histology and immunohistochemistry. Dermal substitutes seeded with DFO-pretreated nanofat exhibited a faster and more extensive vascularization, as evidenced by a significantly higher functional microvessel density in both implant border and center zones when compared to controls. Most blood-perfused microvessels originated from the GFP^+^ DFO-pretreated nanofat. The improved vascularization was associated with reduced leukocyte-endothelial cell interactions in peri-implant venules as well as a decreased implant infiltration by macrophages and neutrophils, indicating an attenuation of the early innate inflammatory response. Moreover, DFO pretreatment promoted the tissue integration of the implants and regenerative extracellular matrix remodeling, as evidenced by increased collagen III deposition. These findings demonstrate that short-term ex vivo DFO pretreatment effectively primes nanofat to enhance microvascular network formation and suppress inflammation, resulting in an accelerated and improved engraftment of nanofat-seeded dermal substitutes.

See also the graphical abstract(Fig. 1).

## Introduction

Dermal substitutes are biomatrices designed to mimic the structure and function of the natural extracellular matrix and dermis with the aim of promoting wound healing and minimizing scarring (Ringrose et al., 2025[31]). They are widely used in plastic and reconstructive surgery amongst other things to manage complex wounds, particularly those resulting from extensive burns or oncologic resections (Campagnari et al., 2017[8]; van den Bosch et al., 2025[39]). Dermal substitutes provide a temporary scaffold that supports cell infiltration, neodermis formation and tissue regeneration, thereby enabling subsequent definitive wound coverage with split-thickness skin grafts (Zhong et al., 2010[48]). Following implantation, they are initially avascular and rely solely on oxygen diffusion and nutrient delivery from the surrounding host tissue, known as graft imbibition (Yasti et al., 2023[47]). Complete vascularization of the implant may require 3 weeks or longer (Frueh et al., 2017[13]). However, as long as the skin physiological barrier is not fully re-established, patients are exposed to a risk of wound infection, extending treatment duration and hospital stay. Accordingly, strategies focusing on accelerating neovascularization of dermal substitutes are of considerable clinical interest (Amirsadeghi et al., 2020[2]).

Recently, nanofat, an adipose tissue-derived product with regenerative properties, has been shown to significantly enhance the vascularization and tissue integration of dermal substitutes implanted into mice (Bonomi et al., 2024[4]). However, functional microvascular networks typically develop only 10 days after implantation. Therefore, novel strategies to further accelerate nanofat-driven vascularization are of particular interest (Bonomi et al., 2025[7]). The vascularization capacity of nanofat is primarily mediated by the interplay of adipose-derived stem cells (ASCs), endothelial cells, microvascular fragments and angiogenic growth factors (Ding et al., 2022[11]; Weinzierl et al., 2022[44]). Among these, ASCs are strongly responsive to hypoxia-inducible factor (HIF)-1α, a key regulator of cellular adaptation to hypoxic microenvironments, which determines angiogenesis, cell survival, and inflammation (Wahl et al., 2016[40]; Zhu et al., 2023[49]). In fact, activation of the HIF-1α pathway induces the transcription of crucial pro-angiogenic mediators, thereby promoting endothelial cell recruitment and vascular sprouting (Shen et al., 2024[32]). Accordingly, targeting the HIF-1α pathway may represent a promising approach to boost the vascularization capacity of nanofat.

Deferoxamine (DFO) is a clinically available, FDA-approved iron chelator with significant potential for tissue regeneration. By inhibiting iron-dependent prolyl hydroxylases, DFO stabilizes HIF-1α and prevents degradation by proteasome, thus mimicking hypoxic signaling even under normoxic conditions (Amraoui et al., 2025[3]). Beyond its well-established pro-angiogenic activity, DFO has shown to modulate both stromal and endothelial compartments via HIF-1α-dependent signaling. It promotes endothelial cell proliferation and migration, while concomitantly attenuating oxidative stress and secondary inflammatory damage in regenerating tissues (Holden and Nair, 2019[15]). Consistent with these pleiotropic effects, DFO has also demonstrated to enhance neovascularization and tissue regeneration across a wide range of preclinical models of ischemic flaps, chronic wounds, diabetic neuropathy, brain injury, bone defects and eventually fat grafting (Wang et al., 2014[41]; Mehrabani et al., 2015[26]; Temiz et al., 2016[36]; Drager et al., 2017[12]; Oses et al., 2017[29]; Wang et al., 2020[42]; Lintel et al., 2022[25]).

Based on these findings, the present study aimed to determine whether short-term ex vivo pretreatment of nanofat with DFO enhances its vascularization and regenerative capacity.

## Materials and Methods

### Animals and housing

All experimental procedures were reviewed and approved by the local authorities (permission number: 19-2024; State Office for Consumer Protection, Saarbrücken, Germany) and were conducted in strict accordance with the European Directive 2010/63/EU, the ARRIVE guidelines and the National Institutes of Health (NIH) Guidelines for the Care and Use of Laboratory Animals (NIH publication No. 85-23, Rev. 1985).

The animals were maintained under standardized laboratory conditions, including a controlled temperature (22 ± 2 °C) and relative humidity (55 ± 10 %), with wood-chip bedding and unrestricted access to water and standard pellet chow (ssniff Spezialdiäten GmbH, Soest, Germany). The animals were either bred in-house at the Institute for Clinical and Experimental Surgery (Saarland University, Homburg, Germany) or obtained from Charles River Laboratories (Sulzfeld, Germany).

To obtain sufficient adipose tissue for nanofat preparation, the inguinal fat pads from eight green fluorescent protein (GFP)^+^ C57BL/6-Tg (CAG-EGFP)131Osb/LeySopJ donor mice of both sexes (age: 6-12 months; body weight: > 30 g; The Jackson Laboratory, Bar Harbor, ME, USA) were harvested. For in vivo analyses, dorsal skinfold chambers were surgically implanted into sixteen GFP^- ^C57BL/6J wild-type recipient mice of either sex (age: 2-6 months; body weight: 22-30 g).

### Anesthesia and perioperative care

For all surgical interventions and intravital microscopic examinations, anesthesia was induced through intraperitoneal injection of ketamine hydrochloride (100 mg/kg body weight; Ketabel^®^, Bela-pharm GmbH & Co. KG, Vechta, Germany) combined with xylazine (12 mg/kg body weight; Rompun^®^, Bayer, Leverkusen, Germany). Perioperative analgesia was achieved through subcutaneous injection of carprofen (10 mg/kg body weight; Rimadyl^®^, Zoetis Deutschland GmbH, Berlin, Germany). To protect the cornea during anesthesia, a lubricating ophthalmic ointment (Bepanthen^®^, Bayer Vital GmbH, Leverkusen, Germany) was applied.

### Nanofat preparation and pretreatment

White subcutaneous adipose tissue obtained from GFP⁺ donor mice was processed into nanofat by a standardized mechanical emulsification, as previously described in detail (Tonnard et al., 2013[38]; Bonomi et al., 2024[4]). Following harvest, the tissue was rinsed in physiological saline and minced into small fragments (approximately 1 × 1 × 1 mm) with a McIlwain Tissue Chopper (CLE Co. Ltd., Gomshall, UK). The minced adipose tissue was subsequently subjected to repeated bidirectional transfer between two syringes connected via sequential female-to-female Luer lock connectors with decreasing inner diameters (2.4, 1.4, and 1.2 mm), with 30 passes performed for each connector size. Following mechanical emulsification, the resulting suspension was passed through a 500 µm mesh filter to eliminate residual larger tissue fragments and debris. The filtered nanofat was then evenly distributed into two microcentrifuge tubes (Eppendorf, Hamburg, Germany) and incubated for 1 h at room temperature in a 1:1 volume ratio with either Hank's Balanced Salt Solution (HBSS; Gibco, Waltham, MA, USA) alone (vehicle control; n = 8) or HBSS supplemented with DFO mesylate (1 mM; Sigma-Aldrich, St. Louis, MO, USA; n = 8).

### Preparation of nanofat-seeded dermal substitutes

The bilayer collagen-glycosaminoglycan-based dermal substitute Integra^®^ (Integra LifeSciences, Ghent, Belgium; thickness: 1.3 mm) was used to generate circular implants with a diameter of 4 mm using a sterile biopsy punch (Kai Europe GmbH, Solingen, Germany). The resulting discs were immediately immersed in either vehicle-pretreated nanofat (control) or DFO-pretreated nanofat and incubated at room temperature for 10 minutes. As previously demonstrated by histological analysis, this short incubation period and seeding technique was sufficient to allow uniform adsorption of the nanofat suspension into the porous matrix of the dermal substitutes, thereby enabling stable loading of the implants (Bonomi et al., 2024[4]).

### Intravital fluorescence microscopy and microvascular analysis

Dermal substitute discs loaded with either vehicle- or DFO-pretreated nanofat were implanted into the observation window of dorsal skinfold chambers in GFP⁻ recipient mice. This experimental setup enabled repeated intravital fluorescence microscopy of the implants at predefined time points following implantation (days 0, 3, 6, 10 and 14). To visualize microvessels and circulating leukocytes, the animals received retrobulbar intravenous injections of fluorescein isothiocyanate (FITC)-labeled dextran (50 µL of a 5 % solution; molecular weight: 150,000 Da; Sigma-Aldrich, Taufkirchen, Germany) for plasma labeling along with rhodamine 6G (50 µL of a 0.1 % solution; Sigma-Aldrich) to stain leukocytes. Fluorescence imaging was performed using an epi-illumination microscope (Axiotech; Zeiss, Oberkochen, Germany) equipped with a monochrome digital camera (Axiocam 702 mono; Carl Zeiss Microscopy GmbH, Oberkochen, Germany). Quantitative image analyses were performed using the CapImage analysis system (version 8.10.1; Dr. Zeintl Software, Heidelberg, Germany). For quantitative evaluation of implant vascularization, eight predefined regions of interest (ROIs) per implant were analyzed, comprising four ROIs at the periphery and four within the center of the implants. Newly formed microvessels were identified based on the presence of red blood cell (RBC) perfusion. The proportion of perfused ROIs was calculated as the percentage of all analyzed ROIs. The functional microvessel density was determined by measuring the cumulative length of RBC-perfused microvessels within each ROI and expressed as cm/cm^2^. In addition, five microvessels per ROI were randomly selected to determine vessel diameter (µm) and centerline RBC velocity (µm/s). These parameters were subsequently used to calculate shear rate (s⁻^1^) and volumetric blood flow (pL/s), as previously described (Koutsiaris et al., 2007[21]). The inflammatory response induced by the implants was assessed in four peri-implant postcapillary or collecting venules. Microhemodynamic parameters, including vessel diameter, centerline RBC velocity, shear rate and volumetric blood flow, were quantified alongside leukocyte-endothelial interactions. Leukocytes were categorized as free-flowing, rolling or adherent. Rolling leukocytes were defined by reduced transluminal velocity and transient endothelial contact, whereas adherent leukocytes were identified by stable attachment to the endothelial surface for a minimum duration of 30s. Leukocyte counts were normalized to the endothelial surface area, which was calculated assuming a cylindrical vessel geometry.

### Histological and immunohistochemical analyses

Following completion of the final intravital microscopy session, the animals were euthanized by administration of an anesthetic overdose followed by cervical dislocation. The dorsal skin encompassing the implanted dermal substitutes was subsequently harvested and processed for histological and immunohistochemical evaluation. Routine hematoxylin and eosin (HE) staining was performed to assess general tissue morphology. In addition, immunohistochemical stainings were carried out to detect endothelial cells (CD31), extracellular matrix components (collagen I and collagen III) and inflammatory cell populations, including T lymphocytes (CD3), macrophages (CD68) and neutrophilic granulocytes (myeloperoxidase, MPO), according to established protocols as described previously (Bonomi et al., 2024[4]).

Microscopic imaging and quantitative analyses were performed using a BX53 microscope (Olympus, Hamburg, Germany) in combination with the cellSens Dimension software (version 1.11; Olympus). Microvessel density was determined by quantifying CD31⁺ vessels within predefined ROIs located at the implant border and center and expressed as the number of vessels per mm^2^. In addition, the fraction of GFP⁺ microvessels was calculated as the percentage of the total vessel count in each group, allowing discrimination between host- and donor-derived vascular structures. Extracellular matrix remodeling was assessed by quantifying collagen I and collagen III content relative to normal skin. Inflammatory cell infiltration was evaluated by counting CD3⁺ lymphocytes, CD68⁺ macrophages and MPO⁺ neutrophils, expressed as cells per mm^2^, in two central and two peripheral ROIs per implant.

### Statistical analysis

The sample size per experimental group (n = 8) was determined based on prior experience with the same experimental model and outcome measures (Pruzzo et al., 2025[30]). No animals were excluded from the analysis and subjects were allocated to experimental groups using random assignment.

All datasets were evaluated for normality and homogeneity of variance using GraphPad Prism software (version 10.1.2; GraphPad Software, San Diego, CA, USA). Depending on the distribution of the data, intergroup comparisons were performed using either an unpaired Student's *t*-test for normally distributed variables or the Mann-Whitney *U* test for non-normally distributed data. Data are presented as mean ± standard error of the mean (SEM), and statistical significance was defined as a two-sided *p* value < 0.05.

## Results

### Intravital fluorescence microscopy

**Figure 2:**
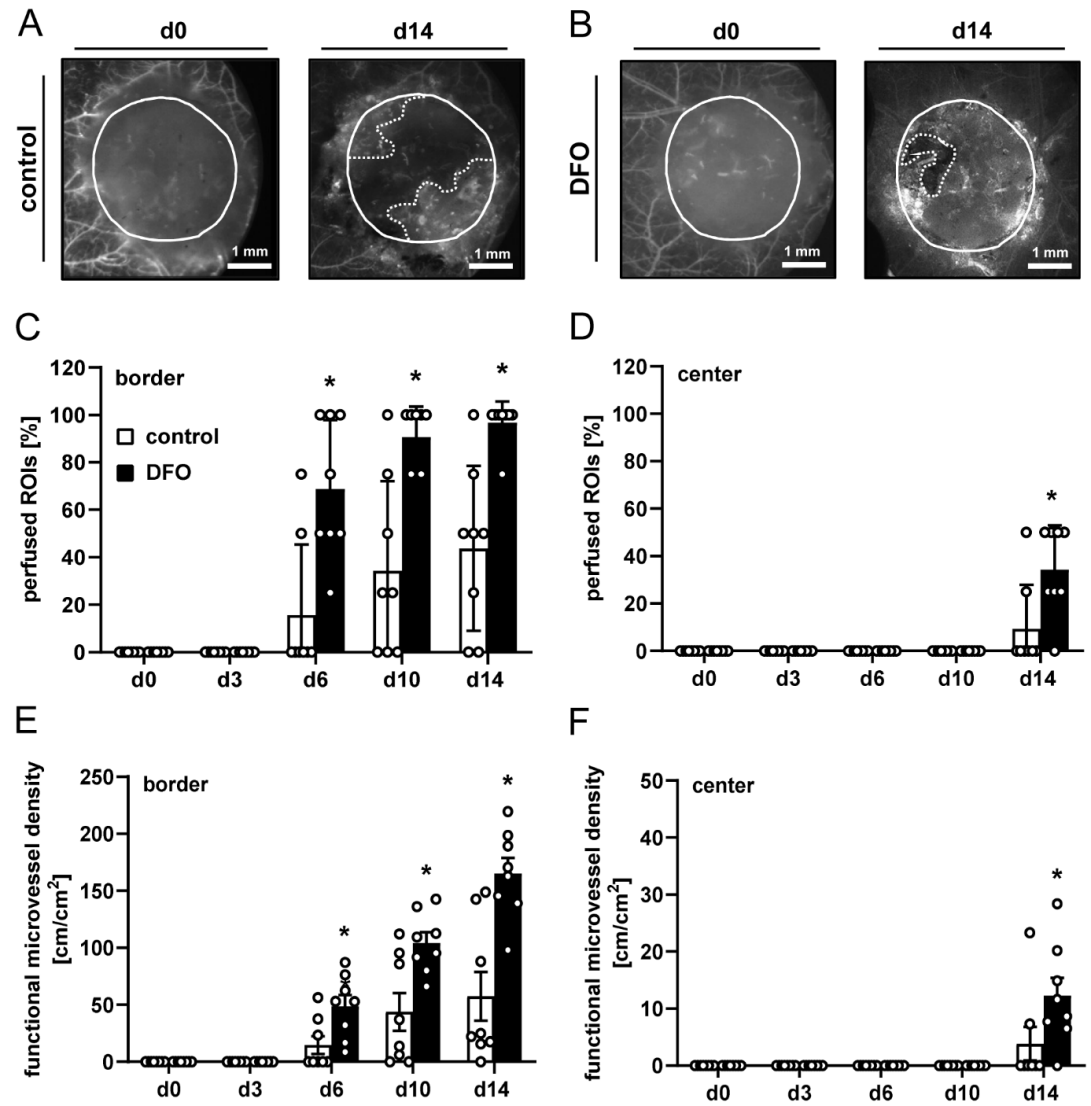
In vivo microscopy of nanofat-seeded dermal substitutes. (A, B) Representative intravital fluorescence microscopic images of dermal substitutes seeded with vehicle-pretreated nanofat (control, (A)) and DFO-pretreated nanofat (DFO, (B)) on day 0 and 14 after implantation into dorsal skinfold chambers of C57BL/6J recipient mice (closed line indicates implant borders; dotted line indicates border of non-vascularized implant area). (C-F) Perfused ROIs (%) (C, D) and functional microvessel density (cm/cm^2^) (E, F) in the border (C, E) and center zones (D, F) of dermal substitutes seeded with vehicle-pretreated nanofat (control; white bars, n = 8) and DFO-pretreated nanofat (DFO; black bars, n = 8). Mean ± SEM; *p < 0.05 vs. control. The raw data are provided in Supplementary Table 1 and Supplementary Table 2.

Repeated intravital fluorescence microscopy enabled the detailed evaluation of the onset of blood perfusion and the progressive development of new microvascular networks in the nanofat-seeded dermal substitutes that had been implanted into dorsal skinfold chambers of recipient mice. Notably, implants seeded with DFO-pretreated nanofat exhibited a much more pronounced microvascular ingrowth both in the border and center zones over time compared to controls (Figure 2A, B(Fig. 2)). This was reflected by a significantly higher number of perfused ROIs and functional microvessel density already at day 6 (Figure 2C-F(Fig. 2)). Microhemodynamic parameters of individual microvessels, such as diameter, centerline RBC velocity, shear rate and volumetric blood flow, were comparable in the two groups (Table 1(Tab. 1)).

**Figure 3:**
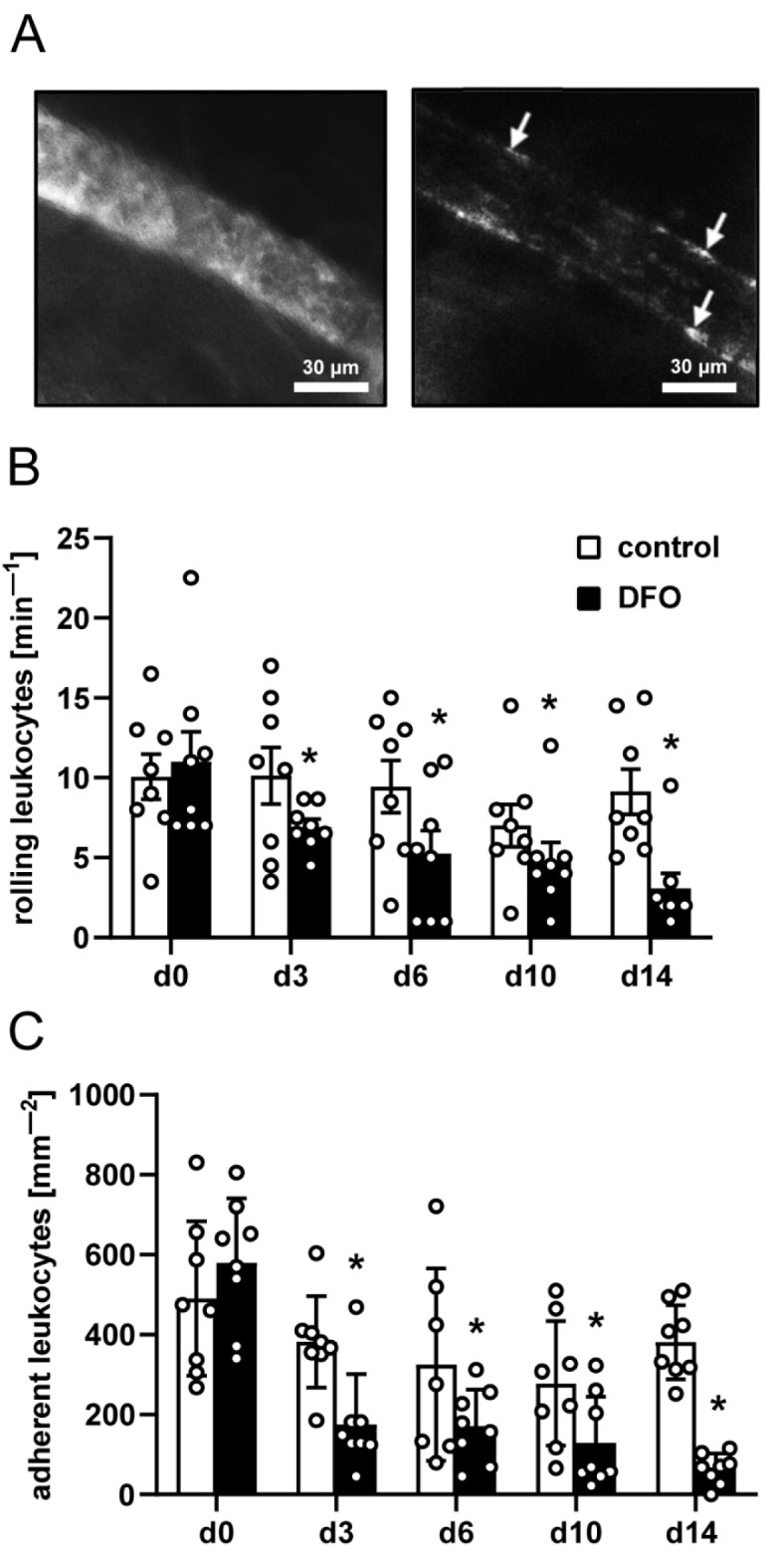
Leukocyte-endothelial cell interactions in response to nanofat-seeded dermal substitutes. (A) Representative intravital fluorescence microscopic images of a collecting venule next to a dermal substitute seeded with vehicle-pretreated nanofat (left panel: blue light epi-illumination, contrast-enhanced by 5 % FITC-labeled dextran; right panel: green light epi-illumination with in situ staining of leukocytes using 0.1 % rhodamine 6G, arrows indicate leukocytes). (B, C) Rolling leukocytes (min^−1^) (B) and adherent leukocytes (mm^−2^) (C) within postcapillary and collecting venules next to dermal substitutes seeded with vehicle-pretreated nanofat (control; white bars, n = 8) and DFO-pretreated nanofat (DFO; black bars, n = 8). Mean ± SEM; *p < 0.05 vs. control. The raw data are provided in Supplementary Table 3.

To evaluate the inflammatory response to the implants, leukocyte-endothelial cell interactions were examined in peri-implant postcapillary or collecting venules of the surrounding subcutaneous host tissue. For this purpose, leukocytes were labeled in situ with 0.1 % rhodamine 6G for their visualization under green-light epi-illumination, while the microvessels were visualized under blue-light epi-illumination following injection of the plasma marker 5 % FITC-labeled dextran (Figure 3A(Fig. 3)). Notably, the number of both rolling and adherent leukocytes was significantly lower on days 3, 6, 10 and 14 in venules around implants seeded with DFO-pretreated nanofat (Figure 3B, C(Fig. 3)). However, these microvessels did not differ in terms of diameter, centerline RBC velocity, shear rate or volumetric blood flow between the two groups, indicating comparable microhemodynamic conditions (Table 2(Tab. 2)).

### Histology and immunohistochemistry

**Figure 4:**
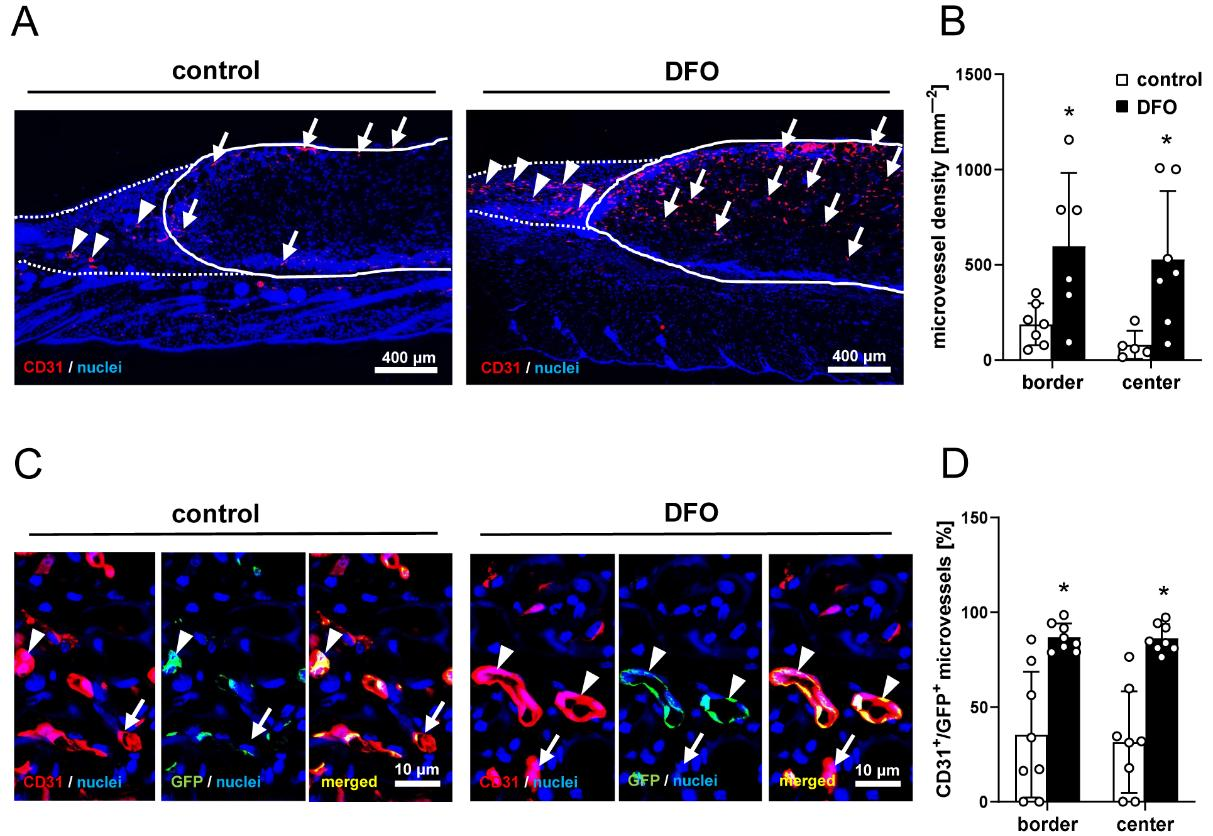
Vascularization of nanofat-seeded dermal substitutes. (A, C) Representative immunohistochemical sections showing CD31^+^ microvessels in the border zones (A, arrowheads) and center zones (A, arrows) as well as the detection of CD31^+^/GFP^−^ (C, arrows) and CD31^+^/GFP^+^ (C, arrowheads) microvessels of dermal substitutes seeded with vehicle-pretreated nanofat (control) or DFO-pretreated nanofat on day 14 after implantation into dorsal skinfold chambers of C57BL/6J recipient mice (closed line indicates implant border; dotted line indicates border zone). (B) Microvessel density (mm^−2^) of dermal substitutes seeded with vehicle-pretreated nanofat (control; white bars, n = 8) and DFO-pretreated nanofat (DFO; black bars, n = 8) on day 14. (D) CD31^+^/GFP^+^ microvessels (%) in the border zones and center zones of dermal substitutes seeded with vehicle-pretreated nanofat (control; white bars, n = 8) and DFO-pretreated nanofat (DFO; black bars, n = 8) on day 14. Mean ± SEM; *p < 0.05 vs. control. The raw data are provided in Supplementary Table 4.

The implanted dermal substitutes were further examined by histology and immunohistochemistry at the end of the in vivo experiments on day 14. The vascularization of the implants was assessed by analyzing CD31-stained sections. Implants seeded with DFO-pretreated nanofat exhibited a markedly higher microvessel density in both the border and center zones compared to controls (Figure 4A, B(Fig. 4)). Furthermore, CD31/GFP co-stainings revealed that more than 90 % of microvessels in this group expressed GFP, indicating that the newly formed vessels originated from the seeded GFP^+ ^nanofat. In contrast, the fraction of GFP^+ ^microvessels was markedly lower in dermal substitutes seeded with vehicle-pretreated nanofat (Figure 4C, D(Fig. 4)).

**Figure 5:**
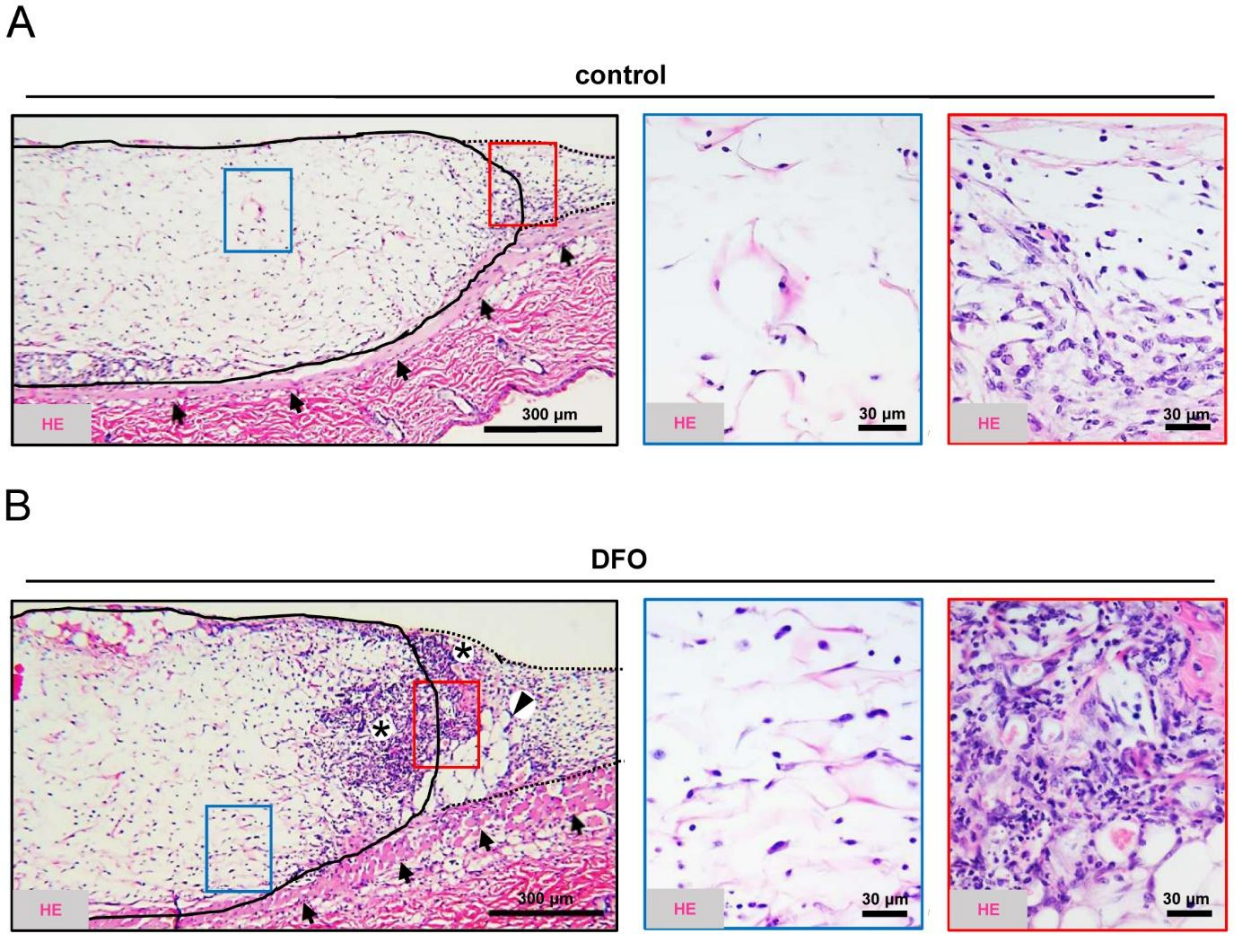
Tissue integration of nanofat-seeded dermal substitutes. (A, B) Representative HE-stained sections of dermal substitutes seeded with vehicle-pretreated nanofat (control, (A)) and DFO-pretreated nanofat (DFO, (B)) on day 14 after implantation into dorsal skinfold chambers of C57BL/6J recipient mice (closed line indicates implant border; broken line indicates border zone; blue and red frames indicate ROIs in the border and center zones of the implants shown in higher magnification on the right panels; arrows indicate panniculus carnosus muscle; asterisks indicate granulation tissue and arrowhead indicates adipocytes).

**Figure 6:**
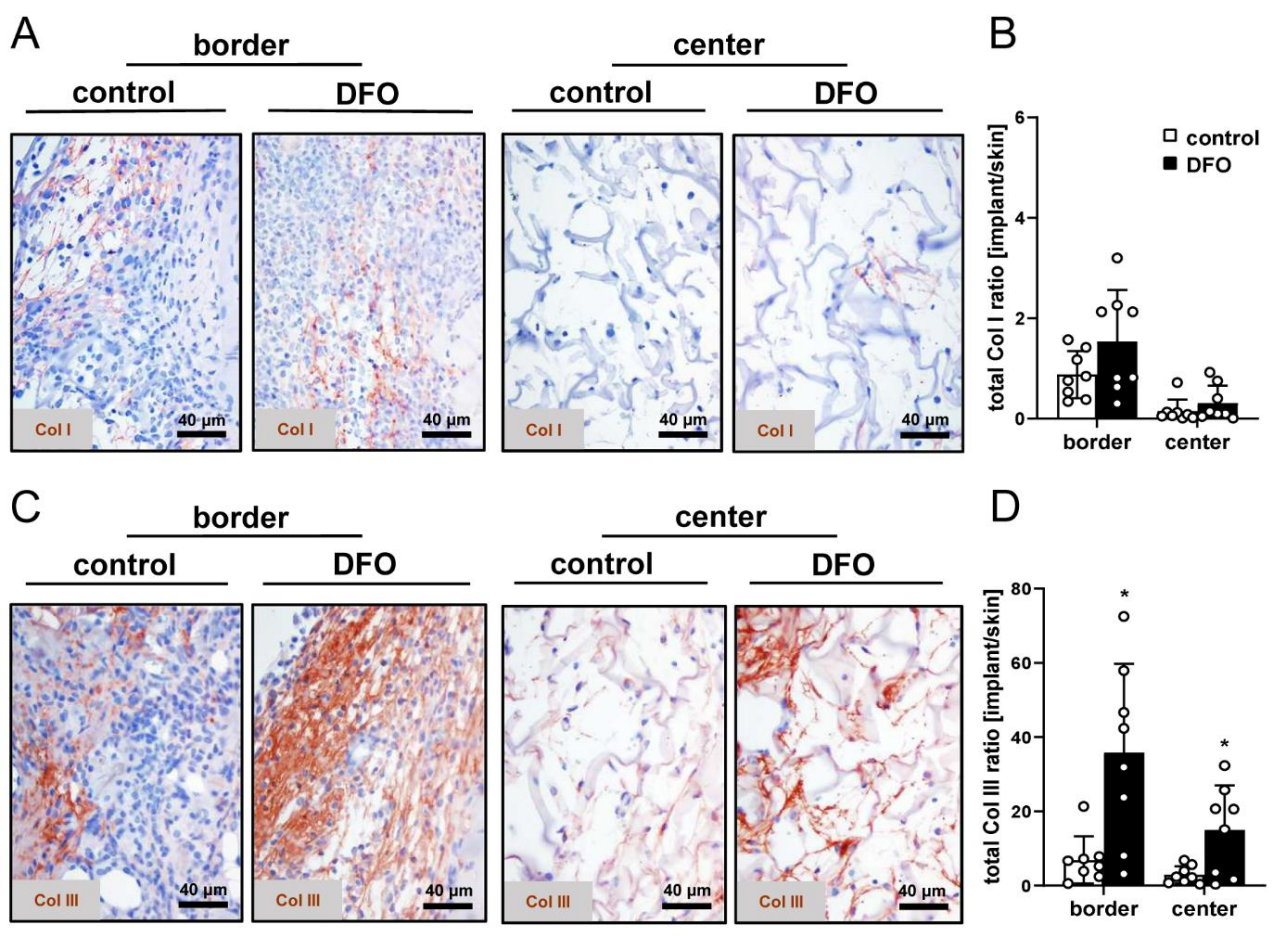
Collagen content in nanofat-seeded dermal substitutes. (A, C) Representative immunohistochemical sections showing Col I (A) and III (C) in the border and center zones of dermal substitutes seeded with vehicle-pretreated nanofat (control) or DFO-pretreated nanofat on day 14. (B, D) Total Col I (B) and Col III (D) ratio (implant/skin) in the border and center zones of dermal substitutes seeded with vehicle-pretreated nanofat (control; white bars, n = 8) and DFO-pretreated nanofat (DFO; black bars, n = 8) on day 14. Mean ± SEM; *p < 0.05 vs. control. The raw data are provided in Supplementary Table 5 and Supplementary Table 6.

The analysis of HE-stained sections revealed an improved tissue integration of dermal substitutes seeded with DFO-pretreated nanofat when compared to controls (Figure 5A, B(Fig. 5)). This was indicated by a more pronounced granulation tissue formation in the border zones of the implants. In addition, these implants contained more residual adipocytes originating from the seeded nanofat (Figure 5B(Fig. 5)). To further investigate tissue integration, the collagen content of the dermal substitutes was quantified, thereby distinguishing between Col I and Col III (Figure 6A-D(Fig. 6)). This analysis revealed a comparable total Col I ratio in the border and center zones of the dermal substitutes in both groups (Figure 6A, B(Fig. 6)). However, the overall Col III ratio was significantly higher in the implants seeded with DFO-pretreated nanofat (Figure 6C, D(Fig. 6)).

**Figure 7:**
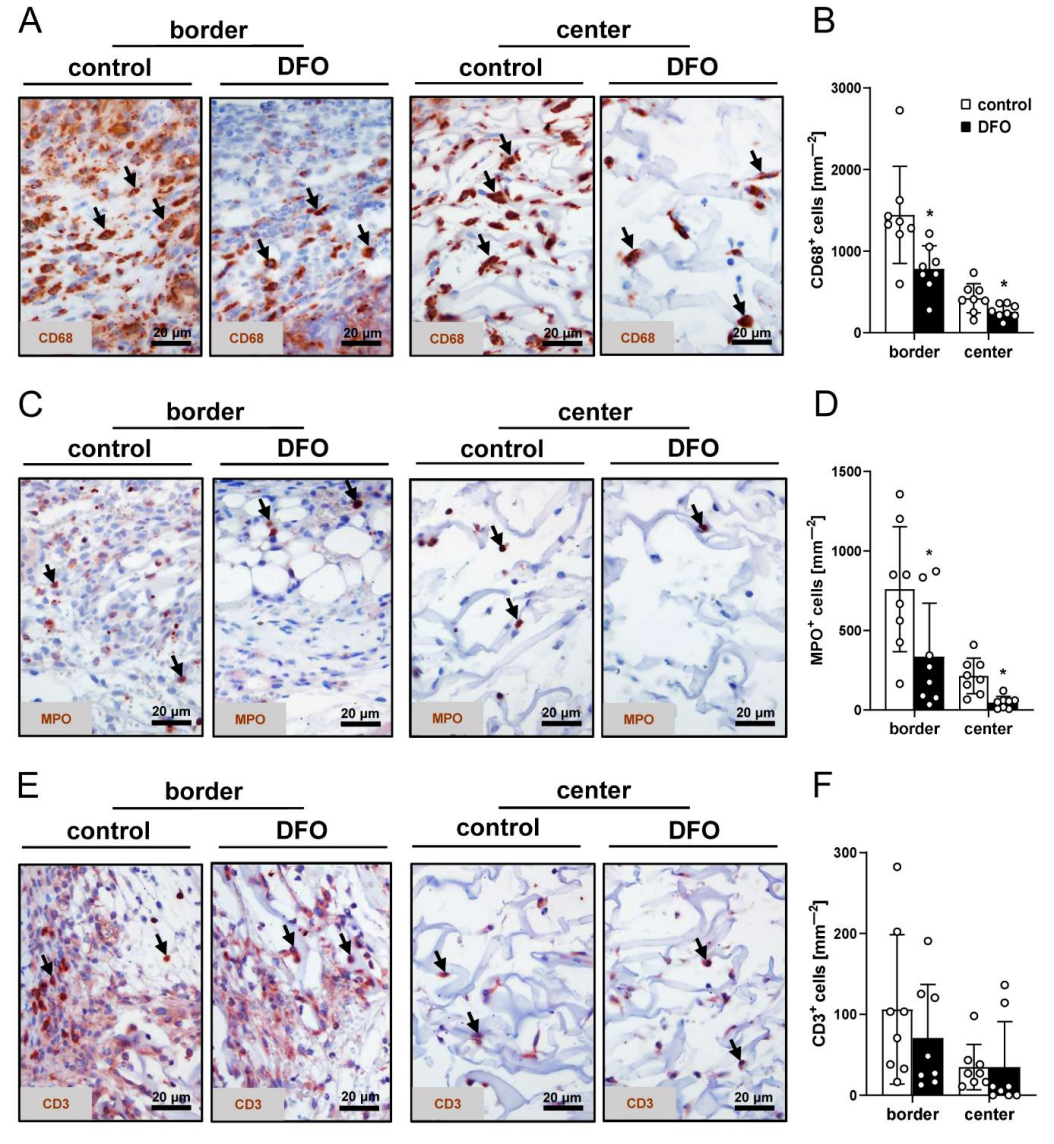
Infiltration of immune cells into nanofat-seeded dermal substitutes. (A, C, E) Representative immunohistochemical sections showing CD68^+^ macrophages (A, arrows), MPO^+^ granulocytes (C, arrows) and CD3^+^ lymphocytes (E, arrows) in the border and center zones of dermal substitutes seeded with vehicle-pretreated nanofat (control) and DFO-pretreated nanofat (DFO) on day 14 after implantation into dorsal skinfold chambers of C57BL/6J recipient mice. (B, D, F) CD68^+^ macrophages (mm^−2^) (B), MPO^+^ granulocytes (mm^−2^) (D) and CD3^+^ lymphocytes (mm^−2^) (F) in the border and center zones of dermal substitutes seeded with vehicle-pretreated nanofat (control; white bars, n = 8) and DFO-pretreated nanofat (DFO; black bars, n = 8) on day 14. Mean ± SEM; *p < 0.05 vs. control. The raw data are provided in Supplementary Table 7.

To assess the immune cell infiltration of the implants, histological sections were stained with antibodies against CD68^+^ macrophages, MPO^+^ neutrophilic granulocytes and CD3^+^ lymphocytes (Figure 7A-F(Fig. 7)). Quantitative analyses of these stained sections showed no marked differences in the lymphocyte content of implants seeded with vehicle- or DFO-pretreated nanofat (Figure 7F(Fig. 7)). However, implants seeded with DFO-pretreated nanofat exhibited a significantly lower density of CD68^+^ and MPO^+^ cells (Figure 7B, D(Fig. 7)), indicating an attenuated early innate inflammatory response.

See also the supplementary information.

## Discussion

Skin reconstruction in extensive or complex wounds using dermal substitutes is a well-established technique, though it still faces major clinical challenges. In fact, a key limiting factor of dermal substitutes is their prompt vascularization and tissue integration after implantation, which are critical preconditions for subsequent successful complete healing. Therefore, strategies that accelerate these processes are of high clinical interest (Bonomi et al., 2025[5]; Pruzzo et al., 2025[30]; Bonomi et al., 2026[6]). The present study demonstrates that short-term ex vivo pretreatment with DFO markedly enhances the vascularization capacity of nanofat. Accordingly, seeding of DFO-pretreated nanofat on dermal substitutes markedly improved their engraftment into the host tissue. This was associated with reduced leukocyte-endothelial cell interactions in peri-implant venules as well as a decreased implant infiltration by macrophages and neutrophils, indicating an attenuation of the early innate inflammatory response.

A major obstacle to the success of tissue-based regenerative therapies, such as the application of nanofat, is the poor survival of transplanted tissues during the early post-implantation period. Tissues maintained under normoxic conditions often fail to adapt to the hypoxic and inflammatory microenvironment of the host tissue, resulting in extensive ischemia-induced cell loss (Hu et al., 2008[17]). Hypoxic preconditioning has therefore been explored to improve post-transplant cell survival and angiogenic activity, primarily by increasing HIF-1α expression (Guo et al., 2022[14]; Wu et al., 2023[46]). This factor induces the transcription of pro-angiogenic mediators, such as vascular endothelial growth factor (VEGF) and stromal cell-derived factor (SDF)-1 (Wlodarczyk et al., 2024[45]). However, the biological consequences of hypoxia are crucially determined by its extent and duration. In this context, we previously demonstrated that severe hypoxic preconditioning of nanofat (1 % O_2_ for 24 h) does not enhance but even impairs its vascularization capacity despite the fact that this intervention upregulates the expression of HIF-1α and SDF-1 within the fat derivative (Bonomi et al., 2026[6]). This apparent paradox underscores a fundamental difference between true hypoxia and pharmacological hypoxia-mimicking. While genuine oxygen deprivation activates HIF-1α signaling, excessive hypoxic stress imposes a substantial metabolic burden, increases the generation of reactive oxygen species and may exhaust cellular adaptive capacity, compromising graft performance after implantation (Alva et al., 2024[1]). In contrast, DFO stabilizes HIF-1α under normoxic conditions. This prevents HIF-1α degradation without exposing cells to actual oxygen deprivation (Mehrabani et al., 2015[26]; Nowak-Stępniowska et al., 2022[27]). Therefore, hypoxia-mimetic signaling enables the accumulation of pro-angiogenic and pro-survival factors while preserving the cellular integrity and metabolic flexibility of the tissue (Janjić et al., 2018[18]; David et al., 2021[9]).

To reduce potential adverse effects, the DFO concentration used in this study was selected based on previous studies. Lin et al. (2023[24]) demonstrated that short-term ex vivo pretreatment of adipose tissue with 1 mM DFO optimally enhances vascularization and graft retention in mice, while avoiding the cytotoxic effects observed at higher concentrations. Across studies, these benefits have been primarily attributed to HIF-1α-dependent upregulation of VEGF signaling and recruitment of endothelial progenitor cells, ultimately promoting rapid vascular ingrowth and graft stabilization (Temiz et al., 2016[36]; Kim et al., 2019[20]; Okyay et al., 2019[28]; Lin et al., 2023[24]). Consistently with these findings, we observed a significantly accelerated and improved vascularization of implanted dermal substitutes seeded with DFO-pretreated nanofat. 

Furthermore, immunohistochemical analyses demonstrated that most newly formed vessels in the implants seeded with DFO-pretreated nanofat were GFP^+^, indicating their origin from the transplanted GFP^+ ^nanofat rather than from ingrowing GFP^-^ microvessels of the surrounding host tissue. This suggests that DFO preserves and enhances the intrinsic vasculogenic capacity of nanofat-resident cellular populations, including endothelial cells and ASCs. In line with this view, HIF-1α activation has shown to directly promote endothelial proliferation, migration and tube formation even under pathological conditions, such as diabetes (Hou et al., 2013[16]; Siavashi et al., 2025[33]). Moreover, HIF-1α upregulates matrix metalloproteinases (MMPs), particularly MMP-2 and MMP-9 (Tajali et al., 2023[35]). These enzymes degrade extracellular matrix components, thereby opening pathways for endothelial cell migration and capillary sprouting. Additionally, MMPs release growth factors, such as VEGF and fibroblast growth factor (FGF)-2, thus amplifying local pro-angiogenic signaling (Oses et al., 2017[29]; Zhu et al., 2023[49]; Tajali et al., 2023[35]).

At the level of extracellular matrix composition, the dense granulation tissue present at the borders of HE-stained implants suggested an improved tissue integration of dermal substitutes seeded with DFO-pretreated nanofat. This was accompanied by a higher number of transplanted adipocytes, indicating improved cell survival and graft integration into the host tissue. Accordingly, additional immunohistochemical analyses revealed a significantly higher collagen III ratio in implants seeded with DFO-pretreated nanofat when compared to controls. However, no significant differences were detected in the collagen I ratio between the two groups. This shift toward collagen III, which predominates during early regenerative wound healing, indicates an active and constructive matrix remodeling phase rather than ischemia-driven matrix stiffening and fibrotic scarring associated with excessive collagen I deposition (Tevlin et al., 2022[37]; Jin et al., 2025[19]; Stewart et al., 2025[34]).

Moreover, DFO pretreatment of nanofat also attenuated the early innate inflammatory response to the implants. This effect is likely multifactorial. By enhancing early perfusion and limiting ischemia-induced tissue injury, DFO reduces the release of damage-associated molecular patterns, thereby attenuating innate immune activation (Li and Nie, 2024[23]). In addition, DFO exerts direct immunomodulatory effects via iron chelation, which reduces the iron-dependent generation of reactive oxygen species and suppresses redox-sensitive inflammatory pathways, including NF-κB signaling, a central regulator of pro-inflammatory cytokine and adhesion molecule expression (Lee et al., 2021[22]). Consistent with this mechanism, previous studies have shown that DFO downregulates key inflammatory mediators, such as intercellular adhesion molecule (ICAM)-1, tumor necrosis factor (TNF)-α and interleukin (IL)-6, and limits macrophage recruitment into inflamed tissues (Wang et al., 2015[43]; Di Paola et al., 2022[10]). The current immunohistochemical results confirmed these findings, demonstrating a reduced infiltration of MPO⁺ neutrophils and CD68⁺ macrophages in implants seeded with DFO-pretreated nanofat, while the amount of CD3^+^ lymphocytes did not differ between the groups. This selective modulation of innate immune cell recruitment, while preserving adaptive immune surveillance, suggests a controlled attenuation of early inflammation that favors angiogenesis and tissue integration. Importantly, this observed anti-inflammatory profile is contradictory to reports of increased inflammation following prolonged or repeated DFO administration (Lee et al., 2021[22]; Di Paola et al., 2022[10]), emphasizing the need for short-term, controlled DFO pretreatment to harness the beneficial effects of this hypoxia mimetic.

Finally, it should be mentioned that the present study also faces some limitations. It was performed in a murine dorsal skinfold chamber model, which enables detailed analyses of early microvascular and inflammatory responses to implanted dermal substitutes but does not fully replicate the complexity of large human wounds. Moreover, the observation period was limited to 14 days and therefore only captures early implant vascularization and tissue integration, while long-term implant performance remains to be determined. Therefore, the validation of our results in clinically relevant large-animal models and pathological wound settings should be the next step towards the translation of our promising novel approach into clinical practice.

## Conclusion

This study demonstrates that short-term ex vivo pretreatment with DFO effectively primes nanofat to enhance microvascular network formation and to suppress inflammation, resulting in an accelerated and improved engraftment of nanofat-seeded dermal substitutes. Of note, nanofat is already routinely used in plastic and reconstructive surgery as an autologous source for soft tissue regeneration. Likewise, DFO is an FDA-approved iron chelator clinically implemented for the management of iron overload disorders, such as hemochromatosis, thalassemia, and myelodysplastic syndrome. Given their established clinical availability and well-characterized safety profiles, the intraoperative pretreatment of nanofat with DFO may represent a translationally feasible strategy to enhance vascularization of dermal substitutes and accelerate soft tissue regeneration.

## Declaration

### Acknowledgments

We are grateful for the excellent technical assistance of Janine Becker (Institute for Clinical and Experimental Surgery, Saarland University, Homburg, Germany). Moreover, the authors thank Servier Medical Art for providing access to designed medical elements (https://smart.servier.com/), supporting the generation of graphical items in this publication.

### Conflict of interest

The authors have no conflicts of interest to declare.

### Artificial Intelligence (AI) - assisted technology

Artificial intelligence was not used in the preparation of this manuscript.

### Author contribution statement

V.P. contributed to the conceptualization of the study, conducted the methodology and investigation, performed formal analysis and data curation, and was responsible for writing - original draft preparation. F.B. contributed to the conceptualization of the study, the methodology, and writing - review and editing. E.L. contributed to the methodology and writing - review and editing. A.W. contributed to the methodology and writing - review and editing. Y.H. contributed to the conceptualization of the study, provided resources, and participated in writing - review and editing. M.W.L. contributed to the conceptualization of the study, the methodology, provided resources, and was responsible for writing - original draft preparation as well as writing - review and editing. All authors have read and agreed to the published version of the manuscript.

### Funding

The present study received no external funding.

## Supplementary Material

Supplementary information

## Figures and Tables

**Table 1:**
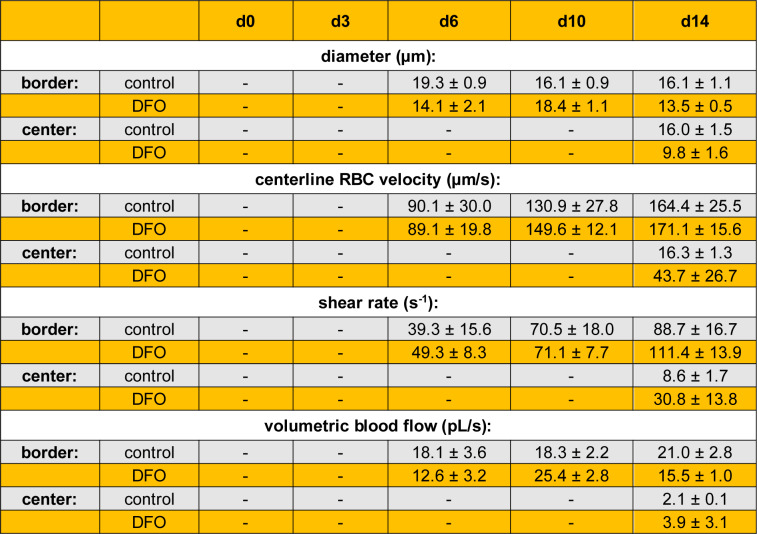
Diameter (µm), centerline RBC velocity (µm/s), shear rate (s^-1^) and volumetric blood flow (pL/s) of microvessels within the border and center zones of dermal substitutes seeded with vehicle-pretreated nanofat (control; n = 8) and DFO-pretreated nanofat (DFO; n = 8). Mean ± SEM; no significant differences

**Table 2:**
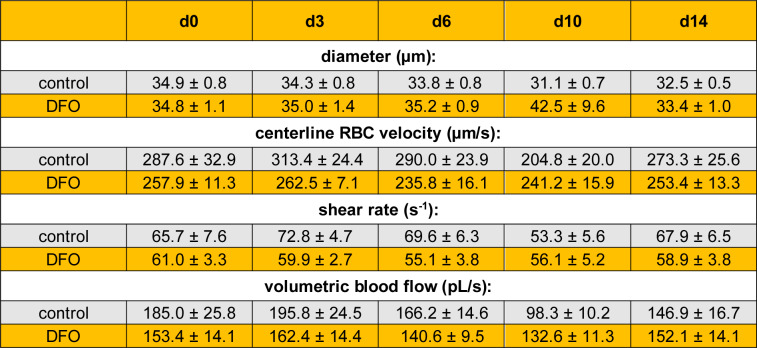
Diameter (µm), centerline RBC velocity (µm/s), shear rate (s^-1^) and volumetric blood flow (pL/s) of postcapillary and collecting venules in direct vicinity to dermal substitutes seeded with vehicle-pretreated nanofat (control; n = 8) and DFO-pretreated nanofat (DFO; n = 8). Mean ± SEM; no significant differences

**Figure 1:**
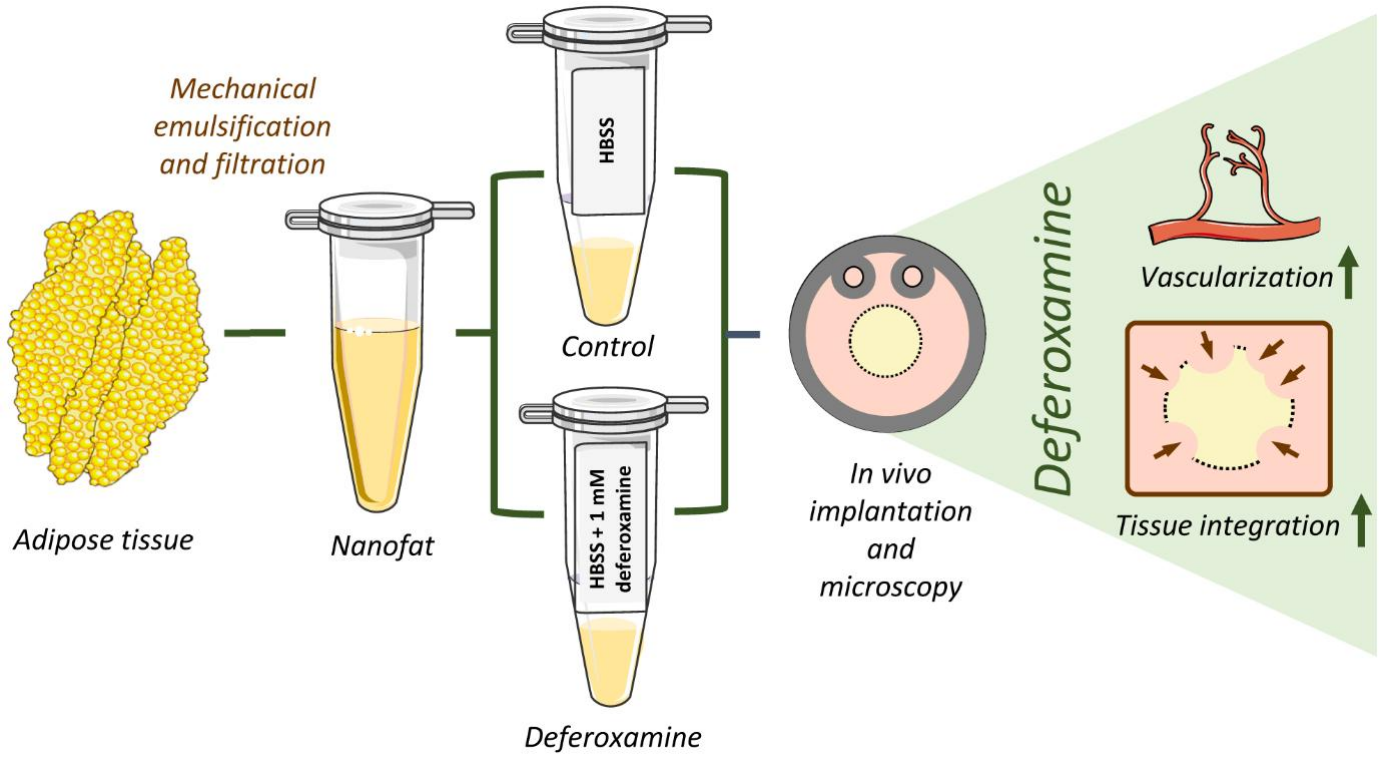
Graphical abstract
