# Supplementary material for: Short-term pretreatment with deferoxamine enhances the in vivo vascularization capacity of nanofat seeded onto dermal substitutes
Source: EXCLI J. 2026 May 7;25:621–37. doi: 10.17179/excli2026-9365 (PMC13247179; doi:10.17179/excli2026-9365)
Supplement: Supplementary information [file EXCLI-25-621-s-001.pdf]

EXCLI Journal 2026;25: 621-637 – ISSN 1611-2156
Received: February 24, 2026, accepted: April 26, 2026, published: May 07, 2026

Supplementary information to:

Original article:

SHORT-TERM PRETREATMENT WITH DEFEROXAMINE ENHANCES THE IN VIVO VASCULARIZATION CAPACITY OF NANOFAT SEEDED ONTO DERMAL SUBSTITUTES

Valeria Pruzzo[1], Francesca Bonomi[1,2], Ettore Limido[1,2], Andrea Weinzierl[1,3], Yves Harder[4,5], Matthias W. Laschke[1*]

[1] Institute for Clinical and Experimental Surgery, Saarland University, PharmaScienceHub (PSH), 66421 Homburg, Germany
[2] Department of Surgery, Ospedale Beata Vergine Mendrisio, Ente Ospedaliero Cantonale (EOC), 6850 Mendrisio, Switzerland
[3] Department of Plastic Surgery and Hand Surgery, University Hospital Zurich, 8006 Zurich, Switzerland
[4] Department of Plastic, Reconstructive, and Aesthetic Surgery and Hand Surgery, Centre Hospitalier Universitaire Vaudois (CHUV), 1011 Lausanne, Switzerland
[5] Faculty of Biology and Medicine, University of Lausanne (UNIL), 1011 Lausanne, Switzerland

* **Corresponding author:** Matthias W. Laschke, MD, PhD, Institute for Clinical and Experimental Surgery, Saarland University, PharmaScienceHub (PSH), 66421 Homburg, Germany; Telephone number: +49-6841-162-6550; E-mail: matthias.laschke@uks.eu

https://dx.doi.org/10.17179/excli2026-9365

This is an Open Access article distributed under the terms of the Creative Commons Attribution License (https://creativecommons.org/licenses/by/4.0/).

EXCLI Journal 2026;25: 621-637 – ISSN 1611-2156
Received: February 24, 2026, accepted: April 26, 2026, published: May 07, 2026

Supplementary Table 1: Perfused regions of interest (ROIs) (%) in the border and center zones of dermal substitutes seeded with vehicle-pretreated nanofat (control; n = 8) and deferoxamine-pretreated nanofat (DFO; n = 8), as assessed in vivo on days (d) 0, 3, 6, 10, and 14 after implantation

Animal	d0	d3	d6	d10	d14
**ROIs border**					
Control 1	0	0	0	25	25
Control 2	0	0	0	50	50
Control 3	0	0	0	0	50
Control 4	0	0	0	25	50
Control 5	0	0	0	0	0
Control 6	0	0	50	75	75
Control 7	0	0	75	100	100
Control 8	0	0	0	0	0
**ROIs center**					
Control 1	0	0	0	0	50
Control 2	0	0	0	0	25
Control 3	0	0	0	0	0
Control 4	0	0	0	0	0
Control 5	0	0	0	0	0
Control 6	0	0	0	0	0
Control 7	0	0	0	0	0
Control 8	0	0	0	0	0
**ROIs border**					
DFO 1	0	0	50	75	100
DFO 2	0	0	50	100	100
DFO 3	0	0	25	75	75
DFO 4	0	0	100	100	100
DFO 5	0	0	75	75	100
DFO 6	0	0	50	100	100
DFO 7	0	0	100	100	100
DFO 8	0	0	100	100	100
**ROIs center**					
DFO 1	0	0	0	0	25
DFO 2	0	0	0	0	25
DFO 3	0	0	0	0	0
DFO 4	0	0	0	0	50
DFO 5	0	0	0	0	50
DFO 6	0	0	0	0	50
DFO 7	0	0	0	0	50
DFO 8	0	0	0	0	25

EXCLI Journal 2026;25: 621-637 – ISSN 1611-2156
Received: February 24, 2026, accepted: April 26, 2026, published: May 07, 2026

Supplementary Table 2: Functional microvessel density (FMD) (cm/cm^2) in the border and center zones of dermal substitutes seeded with vehicle-pretreated nanofat (control; n = 8) and deferoxamine-pretreated nanofat (DFO; n = 8), as assessed in vivo on days (d) 0, 3, 6, 10, and 14 after implantation

Animal	d0	d3	d6	d10	d14
**FMD border**					
Control 1	0.0	0.0	37.7	95.3	88.0
Control 2	0.0	0.0	0.0	6.2	17.6
Control 3	0.0	0.0	0.0	37.1	22.3
Control 4	0.0	0.0	0.0	0.0	15.8
Control 5	0.0	0.0	56.3	86.0	142.7
Control 6	0.0	0.0	0.0	13.6	25.0
Control 7	0.0	0.0	23.4	112.2	148.9
Control 8	0.0	0.0	0.0	0.0	0.0
**FMD center**					
Control 1	0.0	0.0	0.0	0.0	0.0
Control 2	0.0	0.0	0.0	0.0	0.0
Control 3	0.0	0.0	0.0	0.0	0.0
Control 4	0.0	0.0	0.0	0.0	0.0
Control 5	0.0	0.0	0.0	0.0	23.3
Control 6	0.0	0.0	0.0	0.0	0.0
Control 7	0.0	0.0	0.0	0.0	7.3
Control 8	0.0	0.0	0.0	0.0	0.0
**FMD border**					
DFO 1	0.0	0.0	32.3	91.8	139.1
DFO 2	0.0	0.0	16.8	109.4	219.5
DFO 3	0.0	0.0	8.6	66.2	98.0
DFO 4	0.0	0.0	76.4	136.1	170.6
DFO 5	0.0	7.7	62.4	112.5	145.5
DFO 6	0.0	7.0	53.1	80.1	198.5
DFO 7	0.0	0.0	52.9	95.3	162.9
DFO 8	0.0	17.0	87.2	142.6	189.3
**FMD center**					
DFO 1	0.0	0.0	0.0	0.0	7.7
DFO 2	0.0	0.0	0.0	0.0	20.2
DFO 3	0.0	0.0	0.0	0.0	0.0
DFO 4	0.0	0.0	0.0	0.0	28.4
DFO 5	0.0	0.0	0.0	0.0	11.6
DFO 6	0.0	0.0	0.0	0.0	6.5
DFO 7	0.0	0.0	0.0	0.0	14.9
DFO 8	0.0	0.0	0.0	0.0	8.7

EXCLI Journal 2026;25: 621-637 – ISSN 1611-2156
Received: February 24, 2026, accepted: April 26, 2026, published: May 07, 2026

Supplementary Table 3: Rolling leukocytes (min^{-1}) and adherent leukocytes (mm^{-2}) within postcapillary and collecting venules next to dermal substitutes seeded with vehicle-pretreated nanofat (control; n = 8) and deferoxamine-pretreated nanofat (DFO; n = 8), as assessed in vivo on days (d) 0, 3, 6, 10, and 14 after implantation

Animal	d0	d3	d6	d10	d14
**Rolling leukocytes**					
Control 1	8.0	17.0	13.0	6.0	7.5
Control 2	7.5	13.5	12.0	5.5	6.5
Control 3	16.5	4.5	8.5	8.5	15.0
Control 4	3.5	15.0	2.0	8.0	7.5
Control 5	13.0	6.0	15.0	14.5	14.5
Control 6	9.0	3.5	13.5	5.0	5.0
Control 7	10.5	10.5	5.5	7.0	11.5
Control 8	12.5	11.0	6.0	1.5	2.3
**Rolling leukocytes**					
DFO 1	14.0	7.5	11.0	12.0	3.5
DFO 2	11.0	6.5	5.5	5.0	2.0
DFO 3	7.0	6.0	10.5	1.0	1.0
DFO 4	7.0	8.7	1.0	4.0	2.0
DFO 5	7.0	8.7	1.0	4.0	2.0
DFO 6	11.5	4.5	7.0	3.0	9.5
DFO 7	22.5	6.5	5.0	4.5	2.0
DFO 8	8.0	7.0	1.0	5.0	2.5
**Adherent leukocytes**					
Control 1	656.2	603.7	721.4	509.6	408.0
Control 2	460.7	351.0	79.6	116.8	494.4
Control 3	305.8	410.8	424.4	207.9	317.7
Control 4	337.3	355.4	132.6	66.3	332.4
Control 5	830.7	381.0	520.0	464.5	509.5
Control 6	268.3	404.8	325.4	327.0	312.9
Control 7	587.1	367.0	276.0	222.2	422.8
Control 8	474.5	185.2	121.4	308.0	252.3
**Adherent leukocytes**					
DFO 1	652.2	468.5	256.5	260.2	70.7
DFO 2	539.3	45.4	45.5	57.4	48.2
DFO 3	370.9	127.7	312.0	22.4	68.2
DFO 4	640.7	148.5	156.4	204.0	104.2
DFO 5	569.7	129.1	227.0	54.5	75.9
DFO 6	805.6	181.4	178.5	45.2	0.0
DFO 7	720.2	124.7	129.4	68.4	26.3
DFO 8	340.7	181.2	69.0	322.8	115.8

EXCLI Journal 2026;25: 621-637 – ISSN 1611-2156
Received: February 24, 2026, accepted: April 26, 2026, published: May 07, 2026

Supplementary Table 4: Microvessel density (mm^{-2}) and $CD31^+/GFP^+$ microvessels (%) in the border and center zones of dermal substitutes seeded with vehicle-pretreated nanofat (control; n = 8) or deferoxamine-pretreated nanofat (DFO; n = 8), as assessed by immunohistochemistry on day 14 after implantation

Animal	Microvessel density border (mm^{-2})	Microvessel density center (mm^{-2})	$CD31^+/GFP^+$ microvessels border (%)	$CD31^+/GFP^+$ microvessels center (%)
Control 1	351.3	377.8	16.3	17.8
Control 2	210.0	59.1	17.2	0.0
Control 3	130.5	73.7	0.0	34.6
Control 4	77.8	14.6	56.2	0.0
Control 5	727.7	375.6	85.6	76.5
Control 6	294.8	205.5	33.9	32.0
Control 7	186.9	325.1	74.4	59.7
Control 8	52.5	41.9	0.0	31.2
DFO 1	423.7	83.6	94.2	83.8
DFO 2	788.5	1008.3	86.5	84.6
DFO 3	93.1	415.7	91.7	97.3
DFO 4	341.2	198.8	98.5	92.0
DFO 5	1156.6	456.7	81.9	94.3
DFO 6	1772.7	1000.9	79.1	81.1
DFO 7	1884.6	1832.9	83.5	76.4
DFO 8	786.3	530.3	78.9	81.2

EXCLI Journal 2026;25: 621-637 – ISSN 1611-2156
Received: February 24, 2026, accepted: April 26, 2026, published: May 07, 2026

Supplementary Table 5: Total collagen (Col) I ratio (implant/skin) in the border and center zones of dermal substitutes seeded with vehicle-pretreated nanofat (control; n = 8) or deferoxamine-pretreated nanofat (DFO; n = 8), as assessed by immunohistochemistry on day 14 after implantation

Animal	Col I ratio border (%)	Col I ratio center (%)
Control 1	0.8	0.0
Control 2	0.8	0.1
Control 3	1.4	0.7
Control 4	1.6	0.1
Control 5	0.4	0.1
Control 6	0.3	0.0
Control 7	1.2	0.1
Control 8	0.5	0.1
DFO 1	3.2	0.0
DFO 2	2.1	0.9
DFO 3	0.8	0.1
DFO 4	0.6	0.1
DFO 5	0.8	0.4
DFO 6	5.4	0.4
DFO 7	0.3	0.0
DFO 8	2.3	0.8

Supplementary Table 6: Total collagen (Col) III ratio (implant/skin) in the border and center zones of dermal substitutes seeded with vehicle-pretreated nanofat (control; n = 8) or deferoxamine-pretreated nanofat (DFO; n = 8), as assessed by immunohistochemistry on day 14 after implantation

Animal	Col III ratio border (%)	Col III ratio center (%)
Control 1	3.9	2.4
Control 2	21.4	3.9
Control 3	7.9	6.9
Control 4	5.3	5.7
Control 5	6.7	2.2
Control 6	7.2	1.1
Control 7	2.5	0.4
Control 8	0.6	0.8
DFO 1	3.2	0.3
DFO 2	46.7	32.4
DFO 3	8.1	1.6
DFO 4	23.7	20.6
DFO 5	57.9	25.8
DFO 6	72.5	15.2
DFO 7	31.9	3.6
DFO 8	42.4	20.7

EXCLI Journal 2026;25: 621-637 – ISSN 1611-2156
Received: February 24, 2026, accepted: April 26, 2026, published: May 07, 2026

Supplementary Table 7: $CD68^+$ macrophages (mm^{-2}), MPO^+ granulocytes (mm^{-2}), and $CD3^+$ lymphocytes (mm^{-2}) in the border and center zones of dermal substitutes seeded with vehicle-pretreated nanofat (control; n = 8) and deferoxamine-pretreated nanofat (DFO; n = 8), as assessed by immunohistochemistry on day 14 after implantation

Animal	$CD68^+$ (mm^{-2})	MPO^+ (mm^{-2})	$CD3^+$ (mm^{-2})
**Border**			
Control 1	1280.2	561.1	16.3
Control 2	1208.2	667.5	202.1
Control 3	1623.5	849.9	103.5
Control 4	599.3	424.9	70.8
Control 5	2723.9	163.4	32.7
Control 6	1326.2	1201.3	282.4
Control 7	1361.9	1356.5	38.1
Control 8	1427.3	849.9	103.5
**Center**			
Control 1	152.5	157.9	10.9
Control 2	528.4	98.1	98.1
Control 3	397.7	283.3	27.2
Control 4	392.2	217.9	38.1
Control 5	408.6	65.4	16.3
Control 6	250.6	190.7	16.3
Control 7	517.5	408.6	43.6
Control 8	735.5	283.3	27.2
**Border**			
DFO 1	719.1	87.2	190.7
DFO 2	1215.9	173.7	24.8
DFO 3	867.7	342.1	12.9
DFO 4	809.1	270.1	120.6
DFO 5	277.8	76.1	48.4
DFO 6	1051.4	871.7	16.3
DFO 7	599.3	833.5	125.3
DFO 8	713.7	32.7	27.2
**Center**			
DFO 1	359.5	5.4	114.4
DFO 2	365.0	59.9	0.0
DFO 3	212.5	59.9	0.0
DFO 4	261.5	54.5	136.2
DFO 5	207.0	16.3	0.0
DFO 6	321.4	49.0	10.9
DFO 7	114.4	119.8	5.4
DFO 8	234.3	5.4	10.9
